# Cerebral Blood Flow and Amyloid-β Interact to Affect Memory Performance in Cognitively Normal Older Adults

**DOI:** 10.3389/fnagi.2017.00181

**Published:** 2017-06-08

**Authors:** Katherine J. Bangen, Alexandra L. Clark, Emily C. Edmonds, Nicole D. Evangelista, Madeleine L. Werhane, Kelsey R. Thomas, Lyzette E. Locano, My Tran, Zvinka Z. Zlatar, Daniel A. Nation, Mark W. Bondi, Lisa Delano-Wood

**Affiliations:** ^1^Research Service, VA San Diego Healthcare System, San DiegoCA, United States; ^2^Department of Psychiatry, University of California, San Diego, La JollaCA, United States; ^3^San Diego State University, University of California, San Diego Joint Doctoral Program in Clinical Psychology, San DiegoCA, United States; ^4^Psychology Service, VA San Diego Healthcare System, San DiegoCA, United States; ^5^Department of Psychology, San Diego State University, San DiegoCA, United States; ^6^Department of Psychology, University of Southern California, Los AngelesCA, United States

**Keywords:** aging, Alzheimer’s disease, cerebral blood flow, amyloid, arterial spin labeling (ASL), positron emission tomography (PET), neuroimaging, memory

## Abstract

Cerebral blood flow (CBF) alterations and amyloid-β (Aβ) accumulation have been independently linked to cognitive deficits in older adults at risk for dementia. Less is known about how CBF and Aβ may interact to affect cognition in cognitively normal older adults. Therefore, we examined potential statistical interactions between CBF and Aβ status in regions typically affected in Alzheimer’s disease (AD) within a sample of older adults from the Alzheimer’s Disease Neuroimaging Initiative (ADNI) study. Sixty-two cognitively normal participants (mean age = 72 years) underwent neuroimaging and memory testing. Arterial spin labeling magnetic resonance imaging was used to quantify CBF and florbetapir PET amyloid imaging was used to measure Aβ deposition. Aβ status (i.e., positivity versus negativity) was determined based on established cutoffs (Landau et al., 2013). The Rey Auditory Verbal Learning Test was used to assess memory. Linear regression models adjusted for age, education, and sex, demonstrated significant interactions between CBF and Aβ status on memory performance. Among Aβ positive older adults, there were significant negative associations between higher CBF in hippocampus, posterior cingulate, and precuneus and poorer memory performance. In contrast, among Aβ negative older adults, there were no significant associations between CBF and cognition. Our findings extend previous CBF studies of dementia risk by reporting interactions between Aβ status and CBF on memory performance in a sample of well-characterized, cognitively normal older adults. Results suggest that differential CBF-cognition associations can be identified in healthy, asymptomatic Aβ positive older adults relative to Aβ negative individuals. Associations between higherCBF and poorer memory among Aβ positive older adults may reflect a cellular and/or vascular compensatory response to pathologic processes whereby higher CBF is needed to maintain normal memory abilities. Findings indicate that CBF and its associations with cognition may have utility as a reliable marker of brain function early in the AD process when interventions are likely to be beneficial.

## Introduction

Cerebral blood flow (CBF) alterations (Bangen et al., 2014) and amyloid-β (Aβ) accumulation (Rodrigue et al., 2012) have been independently linked to increased risk of developing dementia. It is well established that Aβ accumulation is an early event in the Alzheimer’s disease (AD) pathological process (Jack et al., 2010, 2013) and there is accumulating evidence of the role of early cerebral vascular dysfunction in AD (Iadecola, 2004; Zlokovic, 2011). This includes disruptions in neurovascular function, which is the normal regulation of CBF by arterioles and the capillary neurovascular unit (Girouard and Iadecola, 2006).

Arterial spin labeling (ASL) is a non-invasive magnetic resonance imaging (MRI) technique in which arterial water is magnetically labeled and used as an endogenous tracer to measure CBF (Detre and Alsop, 1999). ASL has been used to reliably measure CBF in AD patients (Johnson et al., 2005); individuals with mild cognitive impairment (MCI) (Bangen et al., 2012); and cognitively normal older adults (Bangen et al., 2009). ASL studies of individuals with AD demonstrate similar patterns of regional hypoperfusion as those shown with studies using fluorodeoxyglucose positron emission tomography (FDG-PET) and single photon emission computed tomography (SPECT) (Chen et al., 2011; Takahashi et al., 2014). ASL techniques have advantages over PET and SPECT including (1) non-invasive use of an endogenous tracer rather than an intravenously administered contrast agent; (2) relatively brief scan times (typically 5–10 min) and can be repeated in short succession due to the magnetization of the labeled blood water that decays within seconds; and (3) quantitative measurement of CBF at rest or during a functional task (Johnson et al., 2005). These advantages along with its increased sensitivity and ability to quantitatively measure perfusion make it ideal to extend its applications for research and in clinical settings (Telischak et al., 2015) designed to monitor neural and vascular changes in healthy aging and disease.

Previous studies have reported associations between Aβ deposition and CBF among older adults across the cognitive spectrum from normal aging to AD. For example, among 182 Alzheimer’s Disease Neuroimaging Initiative (ADNI) participants, Mattsson et al. (2014) reported that higher cortical Aβ load measured by florbetapir PET imaging was associated with reduced CBF in several regions of interest, independent of diagnostic group (cognitively normal, early MCI, late MCI, or AD) (Mattsson et al., 2014). Further, they reported that associations of Aβ load with CBF and brain volume varied across the disease stages. Specifically, in normally aging participants, higher Aβ load was associated with reduced CBF; however, in individuals with late MCI and dementia, higher Aβ load was related to greater reductions of gray matter volume. Given these findings, it was hypothesized that Aβ pathology may lead to reduced CBF early in the disease process and volumetric changes later in the disease process, although longitudinal studies are needed to confirm these temporal relationships (Mattsson et al., 2014). In another study including a sample of 27 cognitively normal older adults and 16 individuals diagnosed with amnestic MCI, Michels et al. (2016) reported a trend toward lower global CBF among those that had greater Aβ deposition measured with Pittsburgh Compound B (PiB) PET (Michels et al., 2016). Taken together, these studies suggest that CBF may be an important mechanism leading to cognitive decline, and may play an even more prominent role among those with elevated Aβ load.

Findings from several postmortem studies (Arriagada et al., 1992; Ingelsson et al., 2004) and *in vivo* PET imaging studies (Engler et al., 2006; Jack et al., 2009) have found no significant association between fibrillar amyloid load and degree of cognitive impairment in individuals with AD dementia. As such, it is thought that fibrillar aggregates of Aβ may not be the immediate cause of cognitive decline and/or Aβ accumulation may be an early event in the AD pathological cascade and may plateau before onset of dementia (Hampel, 2013). If Aβ accumulation is most dynamic before onset of dementia, its effects on cognition should be studied prior to the onset of significant cognitive decline (Hampel, 2013). Although several postmortem and amyloid PET studies have shown that a considerable portion of asymptomatic older adults have increased Aβ burden in the absence of any cognitive impairment (Price and Morris, 1999; Fagan et al., 2006), other previously published reports have shown statistically significant associations between increased Aβ load on PET and poorer cognitive performance in cognitively normal older adults (Rentz et al., 2011). These effects may be best detected on challenging episodic memory tasks and may interact with various AD risk factors such as genetic risk (Pike et al., 2011; Kantarci et al., 2012). Little is known about how CBF and Aβ, which may both serve as early markers of AD changes, may interact to affect cognition in cognitively normal older adults.

There is growing evidence supporting the notion that ASL MRI may be a useful biomarker in predicting cognitive decline and progression to MCI and dementia (Chao et al., 2010; Beason-Held et al., 2013). However, most previous studies have focused on individuals already demonstrating cognitive impairment (MCI and AD) and, to our knowledge, no study has considered how ASL MRI CBF and Aβ status may interact to affect cognition in cognitively normal older adults. Therefore, we examined potential statistical interactions of ASL MRI CBF and Aβ status on cognitive function within a sample of normally aging older adults drawn from the ADNI study.

## Materials and Methods

### The ADNI Dataset

Data used in the preparation of this article were obtained from the ADNI database^1^. The ADNI was launched in 2003 as a public–private partnership, led by Principal Investigator Michael W. Weiner, MD. The primary goal of ADNI has been to test whether serial MRI, PET, other biological markers, and clinical and neuropsychological assessment can be combined to measure the progression of MCI and early AD.

### Participants

Participants were cognitively normal older adults from the ADNI-2 ASL substudy. All participants included in ADNI-2 were between the ages of 55 and 90 years old, had completed at least 6 years of education, were fluent in Spanish or English, and were free of any significant neurological disease other than AD. ADNI control participants had Mini-Mental Status Examination scores ≥ 24 and Clinical Dementia Rating score of 0. Full criteria for ADNI eligibility and diagnostic classifications are described in detail at http://www.adni-info.org/Scientists/ADNIGrant/ProtocolSummary.aspx. This study was approved by the Institutional Review Boards of all of the participating institutions. Informed written consent was obtained from all participants at each site.

Of the 80 control participants who underwent ASL scanning, we included those individuals who had processed data available for download as of September 2016. We further excluded individuals who failed the ADNI raw quality control assessment of ASL data (*n* = 6), were missing PET data (*n* = 1), or were classified as normal controls in ADNI but met criteria for MCI according to comprehensive neuropsychological criteria that operationalizes impairment as performance falling greater than one standard deviation below normative expectations on at least two measures within a cognitive domain (*n* = 11) (Jak et al., 2009; Bondi et al., 2014; Edmonds et al., 2015). This resulted in a final sample of 62 individuals for statistical analyses. The following six measures of cognition were used when diagnosing and excluding for MCI using comprehensive neuropsychological criteria: (1) Animal Fluency, total score; (2) 30-item Boston Naming Test (BNT) total score; (3) Trail Making Test, Part A; time to completion, (4) TMT, Part B; time to completion, (5) Rey Auditory Verbal Learning Test (AVLT) 30-min delayed free recall; number of words recalled, and (6) AVLT recognition; number of words correctly recognized. These measures were selected given their frequent use in assessing early cognitive changes in AD, they were administered to all participants, and they assessed three different domains of cognition – language (Animal Fluency, BNT), speed/executive function (Trail Making Test, Parts A and B), and episodic memory (AVLT recall and recognition).

### Memory Variable Construction

The AVLT assesses an individual’s abilities to acquire 15 words across five immediate learning trials, to recall the words immediately after an intervening interference list (Trial 6), and to recall and recognize the words after a 30-min delay. On verbal serial list-learning tasks, individuals with AD often show a profile involving rapid forgetting after the introduction of an interference trial and profligate responding to delay recognition foils such that overall performance is often at the level of chance (Libon et al., 2011). As such, in addition to AVLT 30-min delayed free recall total number of words recalled and recognition total hits, we calculated additional memory variables to more accurately capture this profile. These additional variables included (1) a post-interference recall score identified as loss of information from Trial 5 to Trial 6 (Mitrushina et al., 1991) and (2) a corrected recognition score considering the number of false positive errors (calculated as [number of recognition hits – number of false positives]). Raw neuropsychological scores for each participant were converted into *z*-scores.

### Arterial Spin Labeling MRI Data Acquisition and Processing

Magnetic resonance imaging was performed on a 3.0 Tesla MR scanners from a single vendor (MAGNETOM Trio, Verio, and Skyra, Siemens). A resting state pulsed ASL scan was acquired utilizing QUIPS II with thin-slice TI1 periodic saturation sequence (“Q2TIPS”) with echo-planar imaging (Luh et al., 1999). The sequence included the following parameters: inversion time of arterial spins (TI1) 700 ms, total transit time of the spins (TI2) 1900 ms, tag thickness 100 mm, tag to proximal slice gap 25.4 mm, repetition time 3400 ms, echo time 12 ms, field of view 256 mm, 64×64 matrix, 24 4 mm thick axial slices [52 tag + control image pairs], time lag between slices 22.5 ms.

Detailed information describing the ASL MRI data acquisition and processing is available online at www.loni.usc.edu. Briefly, the pipeline involves motion correction, aligning each ASL frame to the first frame using a rigid body transformation, and least squares fitting using SPM8. Perfusion weighted images are computed as the difference between the mean of tagged and untagged ASL data sets. Perfusion weighted images were intensity scaled in order to account for signal decay during acquisition and to allow for intensities in meaningful physiological units. After geometric distortion correction, ASL images were aligned to structural T1-weighted images. Given that we are interested in CBF in gray matter and therefore want to minimize the effects of the lower perfusion in white matter on our CBF estimates, a partial volume correction was performed that assumes that CBF in gray matter is 2.5 times greater than in white matter. The partial volume corrected perfusion weighted images were normalized by the reference image (i.e., an estimate of blood water magnetization) to convert the signal into physical units (mL/100 g tissue/min). Quality control procedures include inspecting image quality and rating quality as pass or fail.

FreeSurfer was used to generate anatomical regions of interest (ROIs) for the CBF data and, for secondary analyses, cortical thickness and volume and data for these ROIs. We examined the following four *a priori* ROIs: (1) hippocampus, (2) posterior cingulate, (3) precuneus, and (4) postcentral gyrus. The first three ROIs were selected because they have been implicated in early AD. These regions are part of the neural network subserving episodic memory function and substantially overlap with the default mode network (Hampel, 2013). It’s thought that lifetime cerebral metabolism associated with default activity may predispose these regions to AD-related alterations including Aβ deposition and disrupted connections with the medial temporal lobe which leads to memory impairment (Buckner et al., 2005). A postcentral ROI was selected to serve as a control region, as we do not expect changes in this region in early AD. Mean CBF corrected for partial volume effects was extracted for each of the four ROIs for each hemisphere separately. Mean CBF for each ROI was calculated by averaging the mean CBF of each hemisphere, with each hemisphere’s contribution to the average weighted by the surface area of the ROI for that hemisphere.

### Florbetapir PET Data Acquisition and Processing

A detailed description of ADNI florbetapir PET imaging data acquisition and processing can be found online^2^. Briefly, florbetapir scans were reviewed for quality control before being co-registered, averaged, reoriented into a standard 160 × 160 × 96 voxel image grid with 1.5 mm cubic voxels, and smoothed to a uniform isotropic resolution of 8 mm full width at half maximum. Structural MR images were skull-stripped, segmented, parcellated using FreeSurfer and subsequently co-registered to each participant’s first florbetapir image.

A florbetapir mean cortical summary standardized uptake value ratio (SUVR) was calculated by averaging across the four main cortical regions (i.e., frontal, anterior/posterior cingulate, lateral parietal, and lateral temporal cortices) and dividing by the mean florbetapir value of the whole cerebellum (white and gray matter). Increased retention of florbetapir is thought to reflect greater cortical Aβ load. Aβ positivity versus negativity was determined using the recommended threshold for cross-sectional florbetapir analyses of 1.11 using the whole cerebellum as the reference region (Clark et al., 2012; Joshi et al., 2012; Landau et al., 2013, 2014). In total, 76% of the sample (*n* = 47) was determined to be Aβ negative, while 24% met criteria for Aβ positivity (*n* = 15).

### Statistical Analyses

Chi-squared analyses were utilized to compare the groups in terms of categorical variables and analysis of variance (ANOVA) was used for continuous variables. Hierarchical linear regressions were performed to determine the main effects and interaction of Aβ status (positive or negative) and CBF ROIs on memory performance. For these hierarchical regression analyses, age, education, and sex were the independent variables entered in block 1; CBF of ROIs and Aβ status were predictors entered in block 2; and the interaction term was entered in block 3. Memory variables served as the dependent variable in all regression models. Separate regression models were run for each of the four *a priori* ROIs.

We ran two sets of secondary analyses. First, we ran secondary analyses using the same hierarchical regression models described above but examining Aβ as a continuous variable (i.e., SUVR for the *a priori* ROIs) rather than as a binary variable (i.e., positive versus negative). Second, we ran additional secondary analyses using the same hierarchical regression models described above but also including APOE genotype (𝜀4 carrier versus non-carrier), pulse pressure (i.e., brachial systolic blood pressure minus diastolic blood pressure), and volume (for hippocampus) or cortical thickness (for posterior cingulate and precuneus) for the *a priori* ROI in addition to the demographic variables on block 1. APOE genotype and pulse pressure, a measure of arterial stiffening, are two AD risk factors that are thought to relate to Aβ accumulation and cerebrovascular functioning (Zlokovic, 2011; Bell et al., 2012; Nation et al., 2015). We also adjusted for volume or cortical thickness of the *a priori* ROI of the CBF variable in the model to minimize the potential influence of structural brain changes on findings.

For all analyses, the sign of the post-interference recall score and the Trail Making Test variables was reversed during calculation of *z*-scores to be consistent with the other neuropsychological measures (i.e., higher scores reflect better performance). One Aβ negative participant was not administered AVLT Trial 6 and, therefore, this individual was not included in statistical analyses examining the post-interference recall score (i.e., Trial 5 minus Trial 6). All analyses were performed using the Statistical Package for the Social Sciences (SPSS) version 23 (SPSS IBM, Armonk, NY, United States).

## Results

### Participant Characteristics

Participant demographics are presented in **Table 1**. The Aβ positive group was significantly older, reported fewer years of education, and had a greater proportion of APOE 𝜀4 carriers in comparison to the Aβ negative group (all *p*-values ≤ 0.004). There were no significant group differences with respect to sex, pulse pressure, and cognitive performances across the language, executive functioning, and memory measures (*p*-values > 0.05*)*. There were also no differences between Aβ positive or negative individuals in terms of CBF in any of the ROIs (*p*-values > 0.05).

**Table 1:** Demographic and neuropsychological characteristics of amyloid negative and amyloid positive groups.

	Amyloid-β negative (*n* = 47)	Amyloid-β positive (*n* = 15)	*F* or *X^2^*	Significance	Effect size
**Demographics**					
Age, years, mean (SD)	70.5 (5.8)	76.6 (6.8)	*F* = 11.6	*p* = 0.001	η_p_^2^ = 0.16
Education, years, mean (SD)	17.0 (2.4)	14.1 (3.1)	*F* = 13.7	*p* < 0.001	η_p_^2^ = 0.19
Sex, M:F, (% female)	18:29 (61.7%)	4:11 (73.3%)	*X*^2^ = 0.7	*p* = 0.41	φ_c_ = 0.10
APOE 𝜀4, +:-, (% +)	12:35 (25.5%)	10:5 (66.7%)	*X*^2^ = 8.4	*p* = 0.004	φ_c_ = 0.37
Pulse pressure, mmHg, mean (SD)	60.5 (16.1)	67.5 (11.5)	*F* = 2.4	*p* = 0.13	η_p_^2^ = 0.04
**Cognitive measures (*z*-score)^∗^ mean (SD)**					
***Language***					
Animal Fluency	0.11 (1.03)	-0.35 (0.83)	*F* = 0.05	*p* = 0.83	η_p_^2^ = 0.001
Boston Naming Test	0.00 (1.05)	0.01 (0.87)	*F* = 2.13	*p* = 0.15	η_p_^2^ = 0.04
***Attention/Executive function***					
Trail Making Test, Part A^∗∗^	0.18 (0.86)	-0.57 (1.21)	*F* = 2.47	*p* = 0.12	η_p_^2^ = 0.04
Trail Making Test, Part B^∗∗^	0.17 (0.91)	-0.52 (1.09)	*F* = 0.99	*p* = 0.32	η_p_^2^ = 0.02
***Memory***					
AVLT Recall Total Correct	0.11 (1.01)	-0.35 (0.93)	*F* = 0.45	*p* = 0.51	η_p_^2^ = 0.008
AVLT Post-interference Recall (Trial 5–Trial 6)^∗∗^	0.07 (0.91)	-0.23 (1.25)	*F* = 0.07	*p* = 0.79	η_p_^2^ = 0.001
AVLT Recognition Total Hits	0.01 (0.98)	-0.04 (1.10)	*F* = 1.02	*p* = 0.32	η_p_^2^ = 0.02
AVLT Recognition Corrected Total (Hits–False Positives)	0.05 (0.87)	-0.17 (1.35)	*F* = 0.68	*p* = 0.41	η_p_^2^ = 0.01

*SD, standard deviation; APOE, apolipoprotein E; AVLT, Rey Auditory Verbal Learning Test.*

*^∗^Results from analysis of covariance (ANCOVAs) comparing the amyloid-β positive and negative groups on cognitive measures adjusted for age, education, and gender.*

*^∗∗^The sign for this score was reversed during calculation of *z*-scores to be consistent with the other neuropsychological measures (i.e., higher scores reflect better performance).*

*One Aβ negative participant was not administered AVLT Trial 6 and, therefore, not included in statistical analyses examining the forgetting after interference variable (i.e., Trial 5 minus Trial 6).*

*Amyloid-β negativity versus positivity was based on the recommended threshold for cross-sectional florbetapir analyses of 1.11 using the whole cerebellum as the reference region.*

*In this cognitively normal sample included in the present paper, the mean SUVR with whole cerebellum (gray and white) as reference region was 1.08 (*SD* = 0.15, range = 0.93–1.70). Among Aβ negative individuals, the mean SUVR was 1.02 (*SD* = 0.05, range = 0.93–1.10). Among Aβ positive individuals, the mean SUVR was 1.27 (*SD* = 0.18, range = 1.11–1.70). In our previously published report of ADNI participants with MCI, in a group of single domain amnestic MCI (*n* = 227) and multiple domain dysexecutive/mixed MCI (*n* = 37), the mean cortical summary SUVRs were 1.24 (*SD* = 0.22, range = 0.84–1.86) and 1.37 (*SD* = 0.24, range = 0.88–1.85), respectively (Bangen et al., 2016). Notably, the dysexecutive/mixed group were more severely impaired (i.e., showed impairment in multiple cognitive domains) relative to the amnestic group. In addition, in the present study, in this subset of cognitively normal older adults from the ADNI cohort, 24% of individuals met the threshold for Aβ positivity. A previously published report showed that 29% of participants with normal cognition, 43% of individuals with early MCI, 62% of the participants with late MCI, and 77% of those with Alzheimer’s disease were A positive on florbetapir PET imaging in the ADNI cohort (Landau et al., 2012).*

### Interaction of Amyloid-β and CBF of AD-Vulnerable Regions on Memory Performance

**FIGURE 1:**
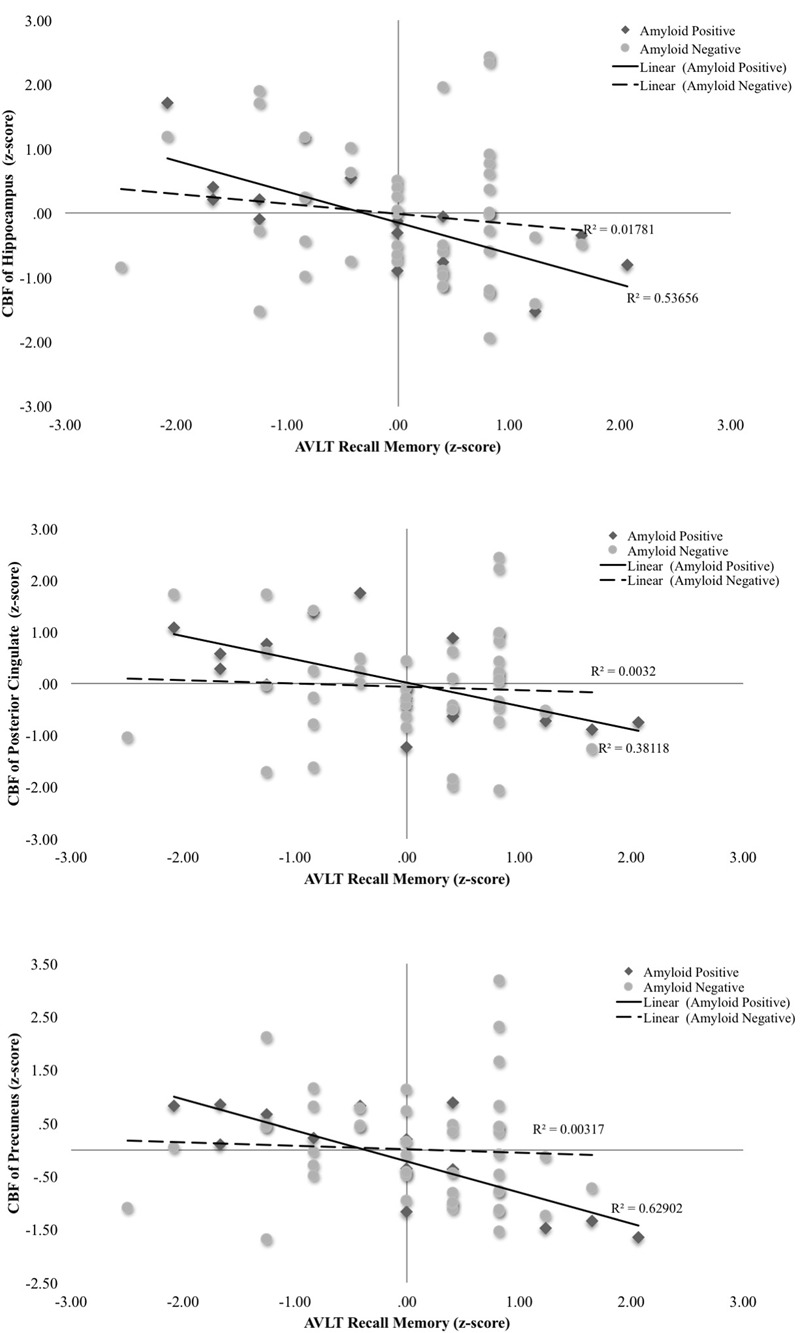
Scatterplots of interaction of Aβ and cerebral blood flow on post-interference recall memory (Rey Auditory Verbal Learning Trial 5-Trial 6 raw *z*-score) for 3 *a priori* cortical regions of interest. Aβ positivity is based on threshold of based on the recommended threshold for cross-sectional florbetapir analyses of 1.11 using the whole cerebellum as the reference region. CBF is presented in standard deviation units. All interactions were statistically significant (*p* < 0.05).

A series of multiple hierarchical linear regression models adjusting for age, education, and sex were first performed to determine whether there was an interaction between Aβ status and CBF of the ROIs on the post-interference recall score (i.e., computed as Trial 5 minus Trial 6). Regression analyses revealed there were significant interactions of Aβ status and CBF in the hippocampus [Δ*F*(1,54) = 8.28, *p* = 0.006, Δ*R*^2^ = 0.11, *B* = -0.11], posterior cingulate [Δ*F*(1,54) = 5.04, *p* = 0.03, Δ*R*^2^ = 0.07, *B* = -0.06], and precuneus [Δ*F*(1,54) = 9.97, *p* = 0.003, Δ*R*^2^ = 0.13, *B* = -0.08]. Examination of simple main effects using non-parametric tests (Spearman’s correlation) revealed there were significant negative associations between post-interference recall memory and CBF of the hippocampus (ρ = -0.78, *p* = 0.001), posterior cingulate (ρ = -0.64, *p* = 0.01), and precuneus (ρ = -0.65, *p* = 0.009) of the Aβ positive group; however, there were no significant associations between post-interference recall memory and CBF of the hippocampus (ρ = -0.14, *p* = 0.37), posterior cingulate (ρ = -0.04, *p* = 0.81), and precuneus (ρ = -0.17, *p* = 0.25) in the Aβ negative group (See **Figure 1** and **Table 2**). When secondary analyses were performed with Aβ as a continuous variable (i.e., SUVR for the *a priori* ROI) rather than as a binary variable (i.e., positive versus negative) regression analyses revealed there was a significant interaction of Aβ and CBF of the precuneus [Δ*F*(1,54) = 6.97, *p* = 0.01, Δ*R*^2^ = 0.10, *B* = -0.18]. Interactions of Aβ and CBF in the hippocampus and posterior cingulate were attenuated and no longer statistically significant [Δ*F*(1,54) = 3.61, *p* = 0.06, Δ*R*^2^ = 0.05, *B* = -0.29] and posterior cingulate [Δ*F*(1,54) = 1.49, *p* = 0.23, Δ*R*^2^ = 0.02, *B* = -0.08].

**Table 2:** Main and interaction effects of amyloid and CBF on post-interference recall.

		Hippocampal CBF					Posterior Cingulate CBF					Precuneus CBF					Postcentral CBF				
Block	Variable	Block *F*	Block Δ*R*^2^	*B* (*SE*)	β	*t*	Block *F*	Block Δ*R*^2^	*B* (*SE*)	β	*t*	Block *F*	Block Δ*R*^2^	*B* (*SE*)	β	*t*	Block *F*	Block Δ*R*^2^	*B* (*SE*)	β	*t*
1	Age	2.25	0.11	-0.04 (0.02)	-0.23	-1.67	2.25	0.11	-0.04 (0.02)	-0.23	-1.67	2.25	0.11	-0.04 (0.02)	-0.23	-1.67	2.25	0.11	-0.04 (0.02)	-0.23	-1.67
	Education			0.06 (0.05)	0.17	1.21			0.06 (0.05)	0.17	1.21			0.06 (0.05)	0.17	1.21			0.06 (0.05)	0.17	1.21
	Sex			0.09 (0.27)	0.05	0.35			0.09 (0.27)	0.05	0.35			0.09 (0.27)	0.05	0.35			0.09 (0.27)	0.05	0.35
2	Aβ (+ versus -)	2.12	0.06	0.04 (0.33)	0.02	0.12	1.68	0.03	0.09 (0.34)	0.04	0.25	1.86	0.04	0.007 (0.34)	0.003	0.02	1.44	0.01	0.08 (0.34)	0.04	0.23
	CBF			-0.03 (0.02)	-0.25	-1.89			-0.01 (0.01)	-0.17	-1.26			-0.02 (0.01)	-0.21	-1.55			-0.01 (0.02)	-0.11	-0.73
3	Aβ × CBF	3.38	0.11	-0.11 (0.04)^∗∗^	-0.37	-2.88	2.34	0.07	-0.06^∗^ (0.03)	-0.31	-2.25	3.46	0.13	-0.08 (0.03)^∗∗^	-0.42	-3.16	1.80	0.05	-0.06 (0.03)	-0.27	-1.81

*^∗^*p* < 0.05, ^∗∗^*p* ≤ 0.01, ^∗∗∗^*p* ≤ 0.001.*

*Dependent variable in all models is post-interference recall (i.e., Rey Auditory Verbal Learning Trial 5–Trial 6).*

*B, unstandardized coefficient estimate.*

*SE, standard error.*

When additional secondary analyses adjusting for APOE genotype (𝜀4 carrier versus non-carrier), pulse pressure, and volume or cortical thickness of the *a priori* ROI were performed, results remained qualitatively and statistically similar to the findings for the primary analyses reported above. There were no main effects of Aβ status or CBF on post-interference recall memory (all *p*-values > 0.05) for any ROI. For delayed recall memory as assessed as total number of words correctly recalled after a 30-min delay, there were no main effects or interactions (all *p*-values > 0.05).

**FIGURE 2:**
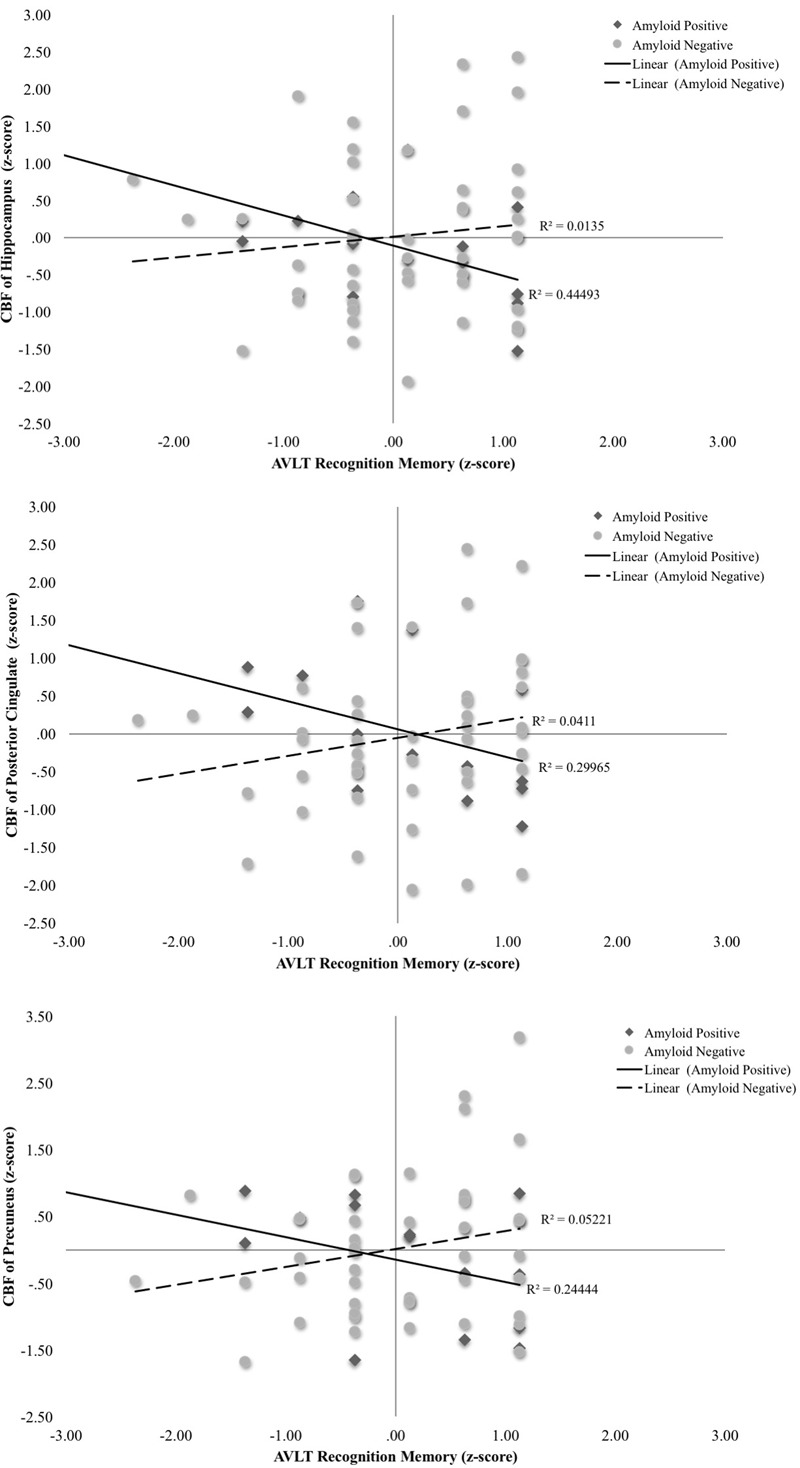
Scatterplots of interaction of Aβ and cerebral blood flow on recognition memory (Rey Auditory Verbal Learning recognition hits-false positives raw *z*-score) for 3 *a priori* cortical regions of interest. Aβ positivity is based on threshold of based on the recommended threshold for cross-sectional florbetapir analyses of 1.11 using the whole cerebellum as the reference region. CBF is presented in standard deviation units. All interactions were statistically significant (*p* < 0.05).

A second set of multiple linear regressions were performed to determine whether there was an interaction between Aβ status and CBF of our ROIs for recognition memory performance (total hits minus false positive errors). Regression analyses adjusting for age, education, and sex, revealed there were significant interactions of Aβ status and CBF in the hippocampus [Δ*F*(1,55) = 11.98, *p* = 0.001, Δ*R*^2^ = 0.15, *B* = -0.13], posterior cingulate [Δ*F*(1,55) = 7.92, *p* = 0.007, Δ*R*^2^ = 0.11, *B* = -0.07], and precuneus [Δ*F*(1,55) = 6.35, *p* = 0.015, Δ*R*^2^ = 0.09, *B* = -0.07]. Examination of simple main effects using non-parametric tests (Spearman’s correlation) revealed there were significant negative associations between recognition memory performance and CBF of the hippocampus (ρ = -0.57, *p* = 0.03) and posterior cingulate (ρ = -0.59, *p* = 0.02) in the Aβ positive group. There was a trend toward worse recognition memory performance and higher CBF of the precuneus in the Aβ positive group (ρ = -0.46, *p* = 0.09). There were no significant associations between recognition memory and CBF of the hippocampus (ρ = 0.13, *p* = 0.39), posterior cingulate (ρ = 0.25, *p* = 0.09), or precuneus (ρ = 0.17, *p* = 0.25) in the Aβ negative group (see **Figure 2** and **Table 3**). When secondary analyses were performed with Aβ as a continuous variable (i.e., SUVR for the *a priori* ROIs) rather than as a binary variable (i.e., positive versus negative), results remained similar. Regression analyses revealed there were significant interactions of Aβ and CBF in the hippocampus [Δ*F*(1,55) = 18.62, *p* < 0.001, Δ*R*^2^ = 0.21, *B* = -0.58], posterior cingulate [Δ*F*(1,55) = 12.69, *p* = 0.001, Δ*R*^2^ = 0.16, *B* = -0.22], and precuneus [Δ*F*(1,55) = 21.13, *p* < 0.001, Δ*R*^2^ = 0.24, *B* = -0.28].

**Table 3:** Main and interaction effects of amyloid and CBF on recognition memory.

		Hippocampal CBF					Posterior Cingulate CBF					Precuneus CBF					Postcentral CBF				
Block	Variable	Block *F*	Block Δ*R*^2^	*B* (*SE*)	β	*t*	Block *F*	Block Δ*R*^2^	*B* (*SE*)	β	*t*	Block *F*	Block Δ*R*^2^	*B* (*SE*)	β	*t*	Block *F*	Block Δ*R*^2^	*B* (*SE*)	β	*t*
1	Age	3.12	0.14	-0.03 (0.02)	-0.23	-1.73	3.12	0.14	-0.03 (0.02)	-0.23	-1.73	3.12	0.14	-0.03 (0.02)	-0.23	-1.73	3.12	0.14	-0.03 (0.02)	-0.23	-1.73
	Education			0.08 (0.05)	0.23	1.72			0.08 (0.05)	0.23	1.72			0.08 (0.05)	0.23	1.72			0.08 (0.05)	0.23	1.72
	Sex			0.12 (0.26)	0.06	0.48			0.12 (0.26)	0.06	0.48			0.12 (0.26)	0.06	0.48			0.12 (0.26)	0.06	0.48
2	Aβ (+ versus -)	1.98	0.01	0.26 (0.33)	0.11	0.79	2.00	0.01	0.27 (0.33)	0.12	0.83	2.13	0.01	0.32 (0.33)	0.14	0.96	2.09	0.01	0.28 (0.33)	0.12	0.86
	CBF			-0.004 (0.02)	-0.04	-0.28			0.004 (0.01)	0.05	0.39			0.01 (0.01)	0.11	0.85			0.01 (0.02)	0.10	0.75
3	Aβ × CBF	3.97	0.15	-0.13 (0.04)^∗∗∗^	-0.43	-3.46	3.17	0.11	-0.07 (0.02)^∗∗^	-0.37	-2.80	3.00	0.12	-0.07 (0.03)^∗^	-0.34	-2.52	2.36	0.06	-0.06 (0.06)	-0.29	-1.81

*^∗^*p* < 0.05, ^∗∗^*p* ≤ 0.01, ^∗∗∗^*p* ≤ 0.001.*

*Dependent variable in all models is recognition corrected for false positive errors (i.e., Rey Auditory Verbal Learning recognition total hits–false positives).*

*B, unstandardized coefficient estimate.*

*SE, standard error.*

When additional secondary analyses adjusting for APOE genotype (𝜀4 carrier versus non-carrier), pulse pressure, and volume or cortical thickness of the *a priori* ROIs were performed, results remained qualitatively and statistically similar to the findings for the primary analyses reported above. There were no main effects of Aβ status or CBF on post-interference recall memory (all *p*-values > 0.05) for any ROI. For delayed recall memory as assessed as total number of words correctly recalled after a 30-min delay, there were no main effects or interactions (all *p*-values > 0.05). In addition, findings were qualitatively and statistically similar when total recognition hits (i.e., not considering false positives) served as the dependent variable. As hypothesized, there were no interactions of Aβ status and postcentral CBF on memory performance (all *p*-values > 0.05). In addition, there were no main effects of Aβ status or CBF on recognition memory performance (all *p*-values > 0.05) for any ROI.

## Discussion

Our study extends previous CBF studies of dementia risk by showing statistical interactions between Aβ status (negative or positive) and regional CBF on memory performance in a sample of well-characterized, cognitively normal older adults. Specifically, we found that among Aβ positive older adults, there were significant associations between higher CBF and poorer verbal memory performance in regions known to be predilections sites for AD—the hippocampus, posterior cingulate, and precuneus. In contrast, among Aβ negative older adults, there were no significant relationships between memory performance and CBF, although there was a trend toward higher CBF in the posterior cingulate and better verbal memory performance. Importantly, our findings demonstrate differential associations between CBF and cognition for Aβ positive versus negative cognitively normal older adults.

Although regional decreases in CBF are interpreted as reflecting decreased brain function, increases in perfusion in the context of preclinical AD—particularly when cognitive performance is maintained or even improved—has often been considered to represent a compensatory response to an incipient pathologic process (Dai et al., 2009). Indeed, several previously published studies have found significant differences in resting hyperperfusion in tandem with better memory function in non-demented older adults at risk for AD, and researchers have interpreted this finding as a potential compensatory response reflecting metabolic alterations and/or increased need for glucose and oxygen to support neuronal activity (Fleisher et al., 2009; Bangen et al., 2012; Zlatar et al., 2014). In contrast, we found that higher resting CBF was associated with *poorer* memory performance among older adults at increased risk for AD by virtue of elevated Aβ accumulation, possibly reflecting cerebrovascular dysregulation or a cellular and/or vascular compensatory response to pathologic processes whereby higher CBF is needed to maintain normal memory abilities. Unlike our previously published work, all individuals in this study were cognitively normal and, importantly, there were no group differences among the Aβ positive and Aβ negative group in terms of cognitive performance. The heightened CBF in Aβ positive individuals may suggest that these individuals are on a declining trajectory of RAVLT performance (albeit still normal), and they need more CBF to support this declining memory system. Hyperperfusion in early MCI followed by hypoperfusion later in MCI when approaching the transition to dementia has been shown and it is possible that the Aβ positive individuals in our sample are closer to developing MCI. Further longitudinal studies investigating perfusion differences across the course of the disease are needed to further examine the role of higher CBF.

Our work showing statistical interactions of perfusion and Aβ status is consistent with previous studies that have demonstrated links between Aβ and cerebrovascular dysregulation. Specifically, prior work has shown that Aβ increases the vulnerability of the brain to cerebral ischemia through its effects on the cells of the neurovascular unit (Zhang et al., 1997; Iadecola, 2004; Girouard and Iadecola, 2006). Moreover, cerebrovascular dysfunction upregulates amyloid precursor protein and Aβ cleavage (Abe et al., 1991; Yokota et al., 1996; Iadecola, 2004). Ultimately, Aβ and cerebrovascular dysfunction are thought to reinforce one another thereby amplifying their deleterious effects on the brain (Iadecola, 2004). The present findings provide further support for the role of vascular alterations in the AD prodrome.

Previous studies of cerebral perfusion across the continuum from the preclinical phase to AD suggest a biphasic pattern characterized by early hyperperfusion preceding later hypoperfusion (Wierenga et al., 2014). In this way, cerebrovascular dysregulation becomes more pronounced over time as the disease progresses (Mentis et al., 1998). This may be due to several factors including neuronal death and synaptic loss resulting in a reduced hemodynamic response to neural activation; accumulating amyloid in cerebral arterioles leading to disruptions in the ability of vascular smooth muscles cells to relax thereby creating a mechanical obstacle to vasodilation (Christie et al., 2001); and atherosclerosis in the circle of Willis (Roher et al., 2003) and conduit cerebral arteries resulting in reduced global CBF and further disruption in the ability of neural stimuli to increase perfusion (Iadecola, 2004). Furthermore, evidence suggests that increased activation within neural networks may modulate Aβ accumulation given that brain regions with lifelong high activity levels (e.g., default mode network) also have the greatest predisposition for Aβ accumulation and increased synaptic transmission results in increased interstitial fluid Aβ levels (Cirrito et al., 2008; Hampel, 2013).

Accumulating evidence suggests that ASL CBF represents a useful biomarker in at-risk individuals since this technique can sensitively differentiate those at risk from control participants (Fleisher et al., 2009; Bangen et al., 2012; Wierenga et al., 2012). Additionally, ASL CBF indices have reliably predicted progression from normal cognition to MCI (Beason-Held et al., 2013), and MCI to AD (Chao et al., 2010). Longitudinal studies have shown that, relative to individuals who remained cognitively normal, older adults who later developed MCI demonstrated hyperperfusion in orbitofrontal, medial frontal, and anterior cingulate regions over time, accompanied by reduced CBF in parietal, temporal, and thalamic regions (Beason-Held et al., 2013). These changes occurred several years prior to the development of cognitive impairment and were observed in regions known to be predilection sites for early AD pathology (Beason-Held et al., 2013). Additionally, these changes were independent of longitudinal changes in tissue volume. This is consistent with findings from our secondary analyses that revealed significant interactions of Aβ status and CBF on memory performance independent of volume or cortical thickness, further suggesting that CBF may play a role in cognitive functioning independent of tissue loss.

In the few existing longitudinal prospective studies using ASL MRI, resting hypoperfusion of the right inferior parietal cortex and right middle frontal cortex at baseline predicted progression from MCI to dementia at 3-year follow-up (Chao et al., 2010) and in another study reduced CBF in the posterior cingulate at baseline was associated with development of cognitive decline at 18-month follow up in healthy older adults (Xekardaki et al., 2015). Our present findings highlight the important association between CBF and memory, and they provide further support for the notion that CBF is a useful marker of AD risk and correlate of cognitive function in older adults. Specifically, we observed evidence of dysregulated CBF patterns in Aβ positive individuals who are cognitively normal suggesting that ASL MRI is sensitive to very early changes in the brain.

The present findings suggest that Aβ accumulation and CBF alterations together influence memory performance in at-risk older adults. These findings add to a growing body of evidence underscoring the importance of multiple pathological processes co-occurring in AD and the interactive influence of several risk factors. Neuropathological studies have shown that clinically diagnosed MCI and AD are both pathologically heterogeneous disorders (Schneider et al., 2007; Nettiksimmons et al., 2014). In our own sample of autopsy-confirmed AD, we found that the presence of mild cerebrovascular changes was associated with less severe AD pathology yet there were no differences in severity of cognitive impairment between the AD patients with and without evidence of cerebrovascular disease (Bangen et al., 2015). These results raise the possibility that cerebrovascular changes contribute to overall severity of cognitive impairment, even in patients with both autopsy-confirmed AD and relatively mild cerebrovascular disease (Bangen et al., 2015). We have also shown that the presence of multiple AD risk factors (e.g., advanced age, APOE 𝜀4 allele, family history of AD, and/or increased vascular risk burden in different combinations) has additive or interactive effects on brain function and cognition (Fleisher et al., 2009; Bangen et al., 2014). The present findings extend this work by demonstrating interactions between PET brain Aβ positivity and CBF on memory performance.

Our results did not reveal any significant main effects of Aβ status or CBF on memory performance in our sample. With respect to Aβ, findings from cross-sectional studies have been inconsistent with some studies reporting no relationship between burden of amyloid in the brain and cognition in cognitively normal or non-demented older adults (Mintun et al., 2006; Aizenstein et al., 2008; Mormino et al., 2009; Rowe et al., 2010) whereas other studies showed associations between greater amyloid and worse cognition (Rodrigue et al., 2012). Additionally, other studies have showed relationships between greater amyloid and worse cognition in APOE 𝜀4 carriers, while no such relationship (Lim et al., 2013) or a weaker relationship among non-carriers (Kantarci et al., 2012).

Prospective longitudinal studies have also been mixed with some studies reporting greater faster rates of cognitive decline in non-demented older adults with high cerebral Aβ load over an 18-month period following PET imaging (Doraiswamy et al., 2012; Lim et al., 2012; Ellis et al., 2013; Kawas et al., 2013) whereas other studies have found no difference in rate of cognitive change over 2- to 3-year follow-up between cognitively normal older adults who had high versus low Aβ at baseline (Villemagne et al., 2011; Ewers et al., 2012). However, prospective longitudinal studies have generally had little follow up after PET imaging (Gu et al., 2015), and retrospective longitudinal studies have shown that non-demented older adults who have higher levels of Aβ showed faster cognitive decline prior to PET scanning relative to their counterparts with lower level of Aβ (Resnick et al., 2010; Landau et al., 2012; Gu et al., 2015). A meta-analysis of 64 studies examining amyloid-cognition associations in healthy older adults found that episodic memory had a small and significant relationship to amyloid burden whereas other cognitive abilities (e.g., working memory, processing speed, visuospatial function, semantic memory) did not have significant relationships to amyloid. Study design, that is cross-sectional vs. longitudinal design, had little influence on findings (Hedden et al., 2013). Although the role of Aβ in cognitive decline and the clinical expression of AD is complex and may be moderated by additional risk factors and variables (Kantarci et al., 2012; Lim et al., 2013; Gu et al., 2015), there is clear evidence to suggest it contributes to the AD process and pivotal to the amyloid cascade model (Jack et al., 2010, 2013).

In contrast to our current ADNI-based findings, we have previously found in our own community samples main effects of CBF on cognition when examining both cognitively normal older adults and those with MCI (Bangen et al., 2012, 2014). In the present sample of ADNI participants, all individuals were cognitively normal and, given selection criteria for ADNI, all participants had very low vascular risk burden. It is possible that we would have found main effects of CBF on memory if there were a greater range of cognitive performance and CBF values. A previously published paper in the ADNI cohort found that the effects of higher brain Aβ load was associated with reduced CBF in cognitively normal older adults and with reduced brain volume in late MCI and dementia suggesting that the relationship between Aβ and CBF changes over the course of the disease (Mattsson et al., 2014). In the current study, we focused on the interaction between Aβ and CBF on memory and it is possible that we would have observed different relationships among Aβ status, CBF, and memory performance if we included participants with more pronounced cerebrovascular disease and/or individuals with MCI or AD. However, given a critical need to examine preclinical AD in its very earliest stages, for the purposes of the current study we emphasized associations among Aβ status, CBF and memory function in older adults who show brain Aβ positivity on PET in the context of no detectable cognitive impairment.

This work has several important research and clinical implications. First, our findings suggest a dynamic relationship between cerebral perfusion and Aβ in the expression of memory function in individuals with preclinical AD. Results further underscore the potential value in examining sensitive vascular variables in the pathogenesis of AD. Additionally, pharmacological and behavioral interventions, including physical exercise, may play a critical role in the regulation of CBF and, ultimately, the prevention of cognitive decline. Interestingly, a recent study showed that older adults taking angiotensin II AT1-receptor blockers exhibited reduced cerebral amyloid retention (Nation et al., 2016). As noted by the authors, this finding is consistent with results from studies in transgenic animals, and they may explain in part why older adults who use AT1-receptor blockers show reduced progression to dementia despite greater vascular risk burden (Nation et al., 2016). Future research is needed to further determine whether anti-hypertension medication and/or behavioral lifestyle changes may improve cerebral microcirculation and reduce Aβ retention.

Strengths of this study include a well-characterized sample of older adults who have undergone multi-modal neuroimaging and neuropsychological assessment as part of a national study on aging and AD. Limitations of our study include use of a global measurement of Aβ pathology rather than local or regional measures. In addition, this was a cross-sectional study and we did not assess cognitive outcome. It is possible that some of the Aβ positive individuals in this study will not develop AD and, likewise, some of the Aβ negative individuals may express the disease at some point. Furthermore, previously published results have reported an absence of cross-sectional associations between amyloid and cognition in healthy controls but have found negative associations for when data is examined longitudinally (Gu et al., 2015). Despite these limitations, in the search for reliable biomarkers of very early AD, ASL MRI may prove especially useful, and the combination of both cerebrovascular and Aβ markers may more completely inform the complex pathological processes underlying the clinical expression of AD than either biomarker class alone. Finally, since vascular risk factors are modifiable, these results may have important implications for biomarker studies, clinical trials, and treatment.

## Author Contributions

KB designed the study, analyzed and interpreted the data, and wrote and revised the manuscript. AC analyzed and interpreted the data and wrote and revised the manuscript. EE, NE, MW, KT, LL, MT, ZZ, DN, MB, and LD-W interpreted the data and revised the manuscript for important intellectual contact. All authors approved the submitted version of the manuscript and agree to be accountable for all aspects of the work.

## Conflict of Interest Statement

The authors declare that the research was conducted in the absence of any commercial or financial relationships that could be construed as a potential conflict of interest.

## Footnotes

1 adni.loni.usc.edu

2 www.loni.usc.edu
